# A perspective from the Mesozoic: Evolutionary changes of the mammalian skull and their influence on feeding efficiency and high‐frequency hearing

**DOI:** 10.1002/ar.25652

**Published:** 2025-03-11

**Authors:** Julia A. Schultz

**Affiliations:** ^1^ Bonner Institute for Organismic Biology – Section Palaeontology Universität Bonn Bonn Germany

## Abstract

The complex evolutionary history behind modern mammalian chewing performance and hearing function is a result of several changes in the entire skeletomuscular system of the skull and lower jaw. Lately, exciting multifunctional 3D analytical methods and kinematic simulations of feeding functions in both modern and fossil mammals and their cynodont relatives approach this topic, giving fresh insights into the history of mammalian masticatory behaviors and their evolutionary trends. One crucial transformation in this context is the segregation of postdentary bones (becoming the mammalian middle ear) from the lower jaw, which is posited to have led to the important functional decoupling of the hearing and feeding systems. Evolution of the middle ear is regarded as the key transition that enhanced both mammalian chewing performance and hearing capacity. Three major functional parts undergo substantial evolutionary changes in this process that are anatomically linked to each other: the lower jaw and dentition, middle ear, and inner ear. Sound, transmitted via vibrations of the bony middle ear elements to the inner ear, is converted into movements of the endolymph fluid that shift hair cells of the organ of Corti, triggering neural stimuli perceived as hearing. Structural changes in one part of the system influence the function of the other two. In this review, I highlight recent advances in research focusing on the enhancement of both chewing performance and hearing ability in mammalian history to feature the mechanisms that led to the decoupling of the hearing system (i.e., middle and inner ear) from the feeding system.

## 1 INTRODUCTION

The ability to efficiently access and digest new food sources is a major driver of adaptive evolution. Mammalian teeth function as tools to break down food via chewing and thus play a key role in food processing. Mammals process their food by mastication, which is the breakdown of food items by repeated occlusal contacts of their teeth during chewing before swallowing (Crompton & Hiiemäe, 1969; Herring, 1993). Physical comminution of the food leads to a larger surface area that can be accessed by digestive bacteria for further biochemical breakdown within the intestines to make nutrients available (Beerton‐Joly et al., 1974; Clauss et al., 2023; Kay & Sheine, 1979; Lumsden & Osborn, 1977; Prinz et al., 2002; Schwermann et al., 2023; Sheine & Kay, 1977). The smaller the particles, the easier the food is digested.

In mammalian evolution, diverse chewing motions evolved as adaptations to different diets (e.g., Bramble, 1978; Herring, 1993; Koenigswald et al., 2013; Ungar, 2010). The broad variety of foods to which mammals are adapted is as versatile as the chewing movements and dental morphologies that exist in this group. Dietary differences range from processing large amounts of green grasses and leaves in large herbivores to meat slicing and bone cracking in carnivores, with many different mixed‐feeding and omnivorous taxa in between to sometimes highly specialized dietary preferences. For example, a large herbivore like the dromedary (*Camelus dromedarius*) utilizes a strong lateral shift of the lower jaw during chewing, with alternating active chewing sides, to break down tough and often dry plant fibers. Being ruminants, dromedaries regurgitate gut content for further physical breakdown by chewing, an evolutionary adaptation to extract even more nutrients from food of low nutrient content. In comparison, small herbivorous mammals like some rodents enhanced the forward (i.e., proal) movement of the lower jaw to use a series of enamel cutting edges on their molars to process seeds or grasses. Carnivorous mammals like wolves (*Canis lupus*) developed sharp carnassials to shear meat off the bones of their prey with a predominantly orthal jaw movement. Between these three extreme examples of jaw motions, there is a wide variety of complex chewing cycles and dietary adaptations in mammals (Lintulaakso et al., 2023).

Chewing function in mammals has a long evolutionary history, as evidenced by dental occlusal features such as directed striations and polished facets on molariforms of Triassic mammaliaforms—the forerunners of mammals, which indicate repeated jaw motions in these early diverging forms (e.g., Crompton, 1971; Jäger et al., 2019; Koenigswald et al., 2013). Diverse dental occlusal features are also known from the rich fossil record of mammalian groups of the Mesozoic, prior to the evolution of complex chewing patterns in extant mammalian groups. In recent years, our knowledge of the evolution of the mammalian feeding apparatus has increased immensely, not only due to a larger number of fossils available but also due to innovative new technology (i.e., 3D analytical methods and kinematic simulations) that facilitated a deeper understanding of the anatomical transformation of the mammalian skull and the functional evolution of feeding and hearing.

The origin of mammals is a key moment in vertebrate history and marks a major transition in vertebrate evolution. This transition is accompanied by fundamental transformations of the skull and jaw, including features for feeding (i.e., jaw and teeth) and sensory function (i.e., sensitive hearing capacity), among a whole suite of other evolutionary innovations (e.g., Allin & Hopson, 1992; Luo, 2011; Luo et al., 2016; Luo & Manley, 2020; Manley & Sienknecht, 2013). During the early stages of mammalian evolution, mandibular bones with both feeding and hearing functions separated from the jaw to become the true mammalian middle ear bones specialized only for hearing (i.e., transmission of airborne sound). The decoupling of the chewing mechanism and hearing function is regarded as a crucial step in mammalian evolution, but there is uncertainty as to when the functional decoupling occurred. For example, evidence for a functional separation of feeding and hearing from the fossil record exists for both early cladotherians (e.g., Bhullar et al., 2019; Grossnickle et al., 2022; Patterson, 1956) and early trechnotherians (e.g., Mao et al., 2020). The separation is hypothesized to have facilitated the efficiency enhancement of both the feeding (i.e., fast and effective food break down) and hearing ability (i.e., reception of wider range of frequencies including high frequencies). The incorporation of multiple bony elements into the middle ear increased the hearing sensitivity of mammals, most notably to high‐frequency sounds, a trait that is thought to have benefited the mammalian lineage (e.g., recent studies of Grossnickle, 2017; Grossnickle et al., 2022; Luo & Manley, 2020; Mao et al., 2020; Mao & Meng, 2020). Thus, the evolution of mammalian hearing and feeding goes hand in hand.

The assumption that most Mesozoic mammals were generalized small animals with generalized feeding habits and a ground‐dwelling (i.e., terrestrial locomotor mode) lifestyle has been refuted by spectacular new findings from the fossil record in the past 25 years (e.g., Luo, 2007; Martin, 2018; Rougier et al., 2011, 2021). Our current view is that the Jurassic and Cretaceous mammal assemblages were ecomorphologically diverse, approaching the diversity of the Cenozoic assemblages of terrestrial, small‐bodied mammals in many aspects (Chen et al., 2019; Luo, 2007; Martin, 2018). Most terrestrial ecological niches and dietary patterns were already established in the extinct mammal groups of the Mesozoic, shaping our view on early mammalian evolution. Besides terrestrial and scansorial adapted animals, fossorial diggers and semi‐aquatic swimmers were discovered, often in stunning preservation (Ji et al., 2006; Luo & Wible, 2005; Mao et al., 2021; Martin et al., 2015). Even gliding non‐mammalian mammaliaforms with the patagium preserved are known from the Jurassic (Meng et al., 2006, 2017). The huge variety of skull and jaw shapes, dental morphologies, and occlusal relationships reflect the broad dietary ecology of mammal relatives from the Mesozoic. Adding to the evidence derived from dental features, their diverse postcranial anatomy (i.e., body and limb bones) shows adaptations to different substrates with different locomotor modes to occupy different niches within a habitat (e.g., Chen & Wilson, 2015; Grossnickle et al., 2019). Almost all diet groups of terrestrial mammals are known from Mesozoic fossils: insectivory, omnivory, carnivory, scavenging, and even feeding on colonial insects convergent to myrmecophagy of some modern mammals (Luo, 2007).

## 2 MAMMALIAN FEEDING EFFICIENCY

The large variety in mammalian cheek tooth (i.e., premolars and molars) morphology reflects adaptation to different diets. Cheek teeth are tools used to shear, crush, or grind food items as effectively as possible in order to extract the maximum amount of energy from the nutrients (Prinz et al., 2002; Prinz & Lucas, 1997). In the chewing process, two relatively static surfaces are initially moved orally toward each other so that food items stuck between the surfaces come under pressure. The different morphologies of tooth surfaces have an influence on the way food is broken down (i.e., shearing, crushing, grinding etc.). Distribution of the pressure acting on the food surface varies with the shape of enamel edges, cusps, and basins. The size of the contact area between food and tooth, with a given force, determines the magnitude of pressure that exceeds the strength of the food and that ultimately leads to breakdown (Agrawal et al., 1997; Evans et al., 2005; Evans & Sanson, 1998; Strait, 1997). The physical properties of the huge variety of food items in the different groups of mammals equally vary immensely. For example, fresh meat is a fibrous and tough medium, not easy to tear with a blunt dentition. Sharp enamel blades are a much better tool to shear meat from bones. In comparison, from soft and juicy leaves or fruits, much more nutrients can be extracted when squeezed and crushed between blunt surfaces. The strong relationship between tooth shape and food adaptation was shown in several studies over the years (see Ungar, 2010, 2015 for detailed overview).

Like the disparity of tooth morphology that is closely linked to diet, the lower jaw shapes of major mammalian clades show disparate morphologies (Fabre et al., 2021; Gill et al., 2014; Grossnickle & Polly, 2013; Morales‐García et al., 2021; Tseng et al., 2023). Recent studies of living and extinct taxa demonstrated that the jaw shapes are correlated with both diet and biomechanical performance (Gill et al., 2014; Grossnickle, 2020a; Morales‐García et al., 2021). On a side note, jaw shape seems also to be influenced by the reproductive mode, with substantial ecomorphological convergence, for example, between metatherians and eutherians, but metatherians generally show lower disparity and a lower rate of evolution (Fabre et al., 2021). Lower jaws are one of the most abundant remnants in the mammalian fossil record besides isolated teeth. For this reason, many studies on mammal evolution focus on fossil jaws and teeth in recent years. In small‐bodied mammals, jaw shape seems to reflect a certain dietary group. This is not only true for modern small mammals but was also demonstrated for non‐mammalian mammaliaforms of the Mesozoic (Morales‐García et al., 2021). 2D geometric morphometrics revealed that insectivorous taxa tend to have long and relatively slender jaws, carnivorous taxa show intermediate to short and stout jaws, while herbivorous taxa tend to have shorter and robust jaws. Correspondence of jaw shape disparity and biomechanical performance has been discussed before by Gill et al. (2014), and more recently by Tseng et al. (2023). Thus, lower jaws of Mesozoic non‐mammalian mammaliaform clades show distinctly disparate morphologies. It was speculated that such major differences not only correlate with the biomechanical performance of the mandible during chewing but also with the evolution and development of the mammalian middle ear (Grossnickle et al., 2022).

The evolutionary changes of the mammalian chewing performance are closely linked to the configuration of the mammalian jaw adductor muscles. It was recently shown that stem therians of the Mesozoic evolved an anteriorly directed jaw movement component, absent in synapsid stem‐line representatives (Grossnickle et al., 2022). To be able to do the anterior shift of the lower jaw, it was necessary to develop anteriorly directed muscle vectors (i.e., general muscle force direction). In cladotherians, this change was accomplished by the appearance of a posteriorly protruding angular process developed at the angle of the lower jaw (Grossnickle, 2017; Patterson, 1956). Anteriorly directed occlusion was very limited in earlier synapsids, likely anatomically hindered by the attached middle ear bones at the posterior part of the lower jaw and lack of a more posteriorly positioned muscle to pull the lower jaw in an anterior direction. Thus, the anatomical shift of the muscle insertion at the posterior end of the lower jaw permitted the evolution of a novel masticatory movement. This was only possible shortly after or directly with the detachment of the middle ear from the lower jaw, which also coincidentally occurs around the cladotherian node, implying that the detachment of the mammalian middle ear of therians likely evolved in the common ancestor of cladotherians. On a side note, the detachment of the middle ear bones from the lower jaw may have evolved independently in multituberculates and monotremes (Fox & Meng, 1997; Luo et al., 2016; Ramírez‐Chaves et al., 2016). It was speculated that the evolutionary shift in the musculature (especially the anterior muscle vector) in connection with the detachment of the middle ear might have been a crucial prerequisite for the dietary diversification of therian mammals (Grossnickle et al., 2022).

Another derived character that appears at the cladotherian node is the unicuspid talonid of the lower molars, a precondition that leads to the development of the tribosphenic molar with a three‐cusped talonid basin in therians. The talonid basin of the tribosphenic molar was shown to have additional occluding areas with shearing potential (Schultz & Martin, 2014), which will later constitute the basis for the extended crushing and shearing functions of the buccal phase II of modern therians after maximum intercuspation.

Ontogenetic age and wear generally seem to influence the chewing performance of mammals (Anders et al., 2011; Ungar, 2015). This relationship is well documented for mostly herbivores with hypsodont dentitions (e.g., Fandos et al., 1993; Damuth & Janis, 2014; Gaillard et al., 2015), but age‐related test series are particularly rare for insectivorous mammals. Schwermann et al. (2023) were able to show that the size of food particles over the lifetime of the small‐bodied insectivorous animal *Tupaia belangeri* does change, with the largest particles in senile individuals, indicating that less precise occlusion due to the wearing down of tooth material with age leads to less precise comminution.

Mammal teeth are worn down over the lifetime; consequently, aged and longer‐used teeth lose important shearing and cutting features with the loss of enamel. Mammal teeth of different wear stages are well known from the fossil record. In particular, in the early history of mammals, the enamel thickness was not as thick or intricately complex in microstructure (i.e., either synapsid columnar or simple plesiomorphic prismatic, with a thick aprismatic outer layer) as in modern mammals (Koenigswald & Sander, 1997; Schultz & Martin, 2011; Wood et al., 1999), indicating a faster collapse of the tooth morphology. According to a stunning recent study of cementum layers by Newham et al. (2020) the lifespan of non‐mammalian mammaliaforms like *Morganucodon* or *Kuehneotherium* was significantly longer than previously thought (i.e., in average 8 to 10 years rather than previously assumed up to 4 years), which correlates very well with the general observation that many fossil remains of these two taxa show extremely worn down molars. Such extreme wear can also be observed in the docodontans and cladotherians from the Jurassic fossil site Guimarota in Portugal.

**FIGURE 1:**
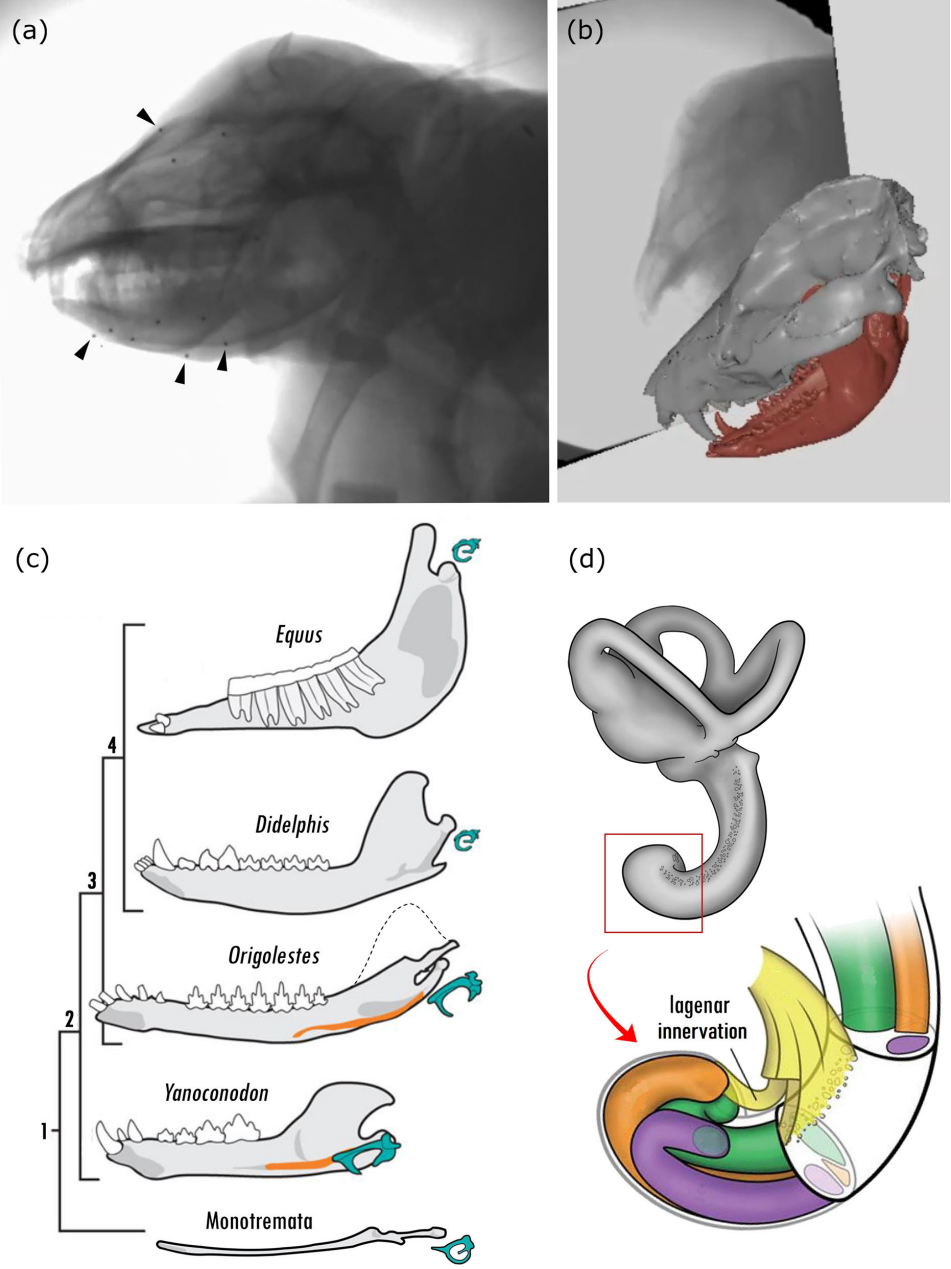
(a) Male opossum (*Didelphis virginana*) eating cheese cubes in the XROMM setting to visualize in vivo chewing movements, black arrow heads indicate tantalum markers that are used to track chewing movements. (b) Reconstructed 3D surface model of the opossum skull aligned with the in vivo movements (video in the background) derived from the XROMM recordings. (c) Key taxa from the mammalian fossil record in a simplified phylogeny of Theria with Monotremata as outgoup (*Equus* and *Didelphis* as representatives of modern placentals and marsupials) illustrating the evolutionary decoupling process of the middle ear ossciles, which segregate from the lower jaw to become the true mammalian middle ear (modified from Schultz, 2020). (d) Line drawing of the inner ear endocast of the platypus (*Ornithorhynchus anatinus*) with multiple openings for the innervation along the curved cochlear canal (gray), red box indicates the apex of the cochlear canal inside which the curved tip of membranous cochlea (zoomed in) is housed, consisting of the membranous compartments scala media (orange), scala tympani (purple), scala vestibuli (green), innervation (yellow) (modified from Schultz et al., 2017). Monotremes show an apical inflation at the end of their curved cochlear canal similar to the morphology found many Mesozoic mammalian ancestors.

In the past years, the chewing biomechanics of early mammalian taxa were studied with 3D visualization methods like the occlusal fingerprint analysis (OFA), finite element analysis (FEA) or multibody dynamics analysis (MDA). More recently, new studies use, for example, the x‐ray of moving morphology (XROMM) approach that records the chewing cycle of living mammals in vivo in stunning detail (Figure 1a), visualizing jaw motions in 3D. However, until now, 3D studies and results are comparably few that allow conclusions on extinct taxa, particularly with a focus on the origin of mammals and chewing behavior in Mesozoic taxa (e.g., Gill et al., 2014; Jäger et al., 2019, 2020; Lautenschlager et al., 2017, 2018; Martin et al., 2020; Schultz et al., 2019; Schultz & Martin, 2014). The research on facet analysis with the aid of OFA simulation proved to be useful in testing existing hypotheses on occlusion and chewing biomechanics, adding new information on life‐ and feeding styles of extinct species and their role within their ecosystems (Martin et al., 2020). With the help of FEA, it was demonstrated that previously hidden trophic specialization at the base of the mammalian radiation occurred, showing that even the earliest non‐mammalian mammaliaform lower jaws were quite diverse morphologically, functionally, and ecologically (Gill et al., 2014).

Adding a fresh view, the XROMM approach provides new discussion points for other 3D kinematic studies by looking into chewing cycle of living mammals in (Figure 1a,b). For example, the hypothesized significance of hemimandible roll (rotation around the long axis of one hemimandible) over jaw yaw (rotation around a dorsoventrally‐oriented axis of the complete jaw) for the tribosphenic molar function in the didelphid *Monodelphis* discussed by Bhullar et al. (2019) could not be fully supported by the study of Stilson et al. (2023) testing chewing cycles of *Didelphis*. Both studies base their working hypotheses on the assumption that the didelphid molar and jaw morphology is suitable to draw conclusions on “early mammalian” chewing patterns and early therian chewing biomechanics and for inference on non‐mammalian mammaliaform chewing performance. The approach to use didelphid marsupials as a baseline comparison started in the 1970s (e.g., Crompton & Hiiemäe, 1969). Crompton et al. (1977) advocated “the Virginian (American) opossum, *Didelphis virginiana* retains many of the craniofacial features of primitive mammals, including a tribosphenic molar and is, therefore, a good structural analogue for the jaw apparatus of early mammals.” This view is debated because of apparent fundamental differences in the structure of the jaws beside the relatively conservative tribosphenic dentition.

Metatherians in general show a significant difference from other mammals in having an inflected angular process. A feature that is known from the fossil record only in the metatherian line appearing at the base when therians split, long after taxa like the morganucodontid *Morganucodon* or the docodontan *Haldanodon* had gone extinct. On a side note, in those two taxa the angular process is rather bent outwards, opposite to the metatherian morphology (“efflected mandibular angle” following Ji et al., 2006 or Schultz et al., 2019) with implications for muscle attachment and biomechanical function during jaw closing. Comparisons seem particularly difficult for major Mesozoic mammal groups that even lack the angular process completely (e.g., spalacotherioids, eutriconodonts, multituberculates). In addition, the didelphid jaw joint is very different from that of the non‐mammalian mammaliaform *Morganucodon*, which has a double jaw joint function with the middle ear bones still attached to the lower jaw. Relating to that, the mandible corpus of *Morganucodon* shows the deep postdentary grooves that occupy the middle ear bones at the posterior end (same as for docodontans) that influence the biomechanical behavior (Lautenschlager et al., 2023; Morales‐García et al., 2019), compared to the derived therian jaw without such grooves. Lastly, the shape and number of teeth in opossums is very different from most non‐mammalian mammaliaform taxa (i.e., pre‐tribosphenic cheek teeth lacking a talonid basin, high number of molars and premolars, and different root implantation pattern). However, it remains to be tested how much of the listed differences change the biomechanical behavior during the chewing process. To be more specific, the lower jaw shape of a didelphid closely represents the primitive condition of early metatherian mammals, but for any comparative statement further down the ancestral line before the split of Metatheria and Eutheria, more testing is needed.

XROMM is certainly a method that will broaden our understanding and knowledge of mammalian chewing performance in vivo in the future. Currently, studies on the chewing performance of small mammals are somewhat rare, and because this method is a relatively new field, those studies sometimes give conflicting results on modern mammal taxa (Bhullar et al., 2019; Stilson et al., 2023). This indicates that assumptions from in vivo studies drawn on early mammalian chewing behavior (especially on non‐mammalian mammaliaform chewing patterns) should be made with caution.

XROMM studies so far (see for example Stilson et al., 2023) show that the mammalian skull and lower jaw have a much more flexible response to strain and stress than previously thought. The high mobility of the symphysis was shown to correlate to different food properties that influence chewing patterns (Stilson et al., 2023). In the context of evolution, this flexibility is surprising, and it is unresolved if the same flexibility occurred in the non‐mammalian mammaliaform chewing performance or if it is purely a cladotherian trait. Such flexibility is less expected or even improbable for non‐mammalian mammaliaforms that had a set of middle ear bones attached to the posterior part of the mandible. This mandibular configuration likely showed a different response to stresses and strains than modern mammalian jaws. The attachment of the middle ear elements certainly influenced the mobility of the middle ear bones, which transmit sound waves via bone conduction from the mandible to the inner ear (Kermack & Mussett, 1983; Luo et al., 2016). The mobility of individual bony middle ear elements is key to the transmission of sound. A larger flexibility of the jaw bone responding to stresses or strains from chewing musculature appears counterintuitive in this context.

## 3 HIGH‐FREQUENCY HEARING IN MAMMALS

The mammalian ear structure is a result of the overall evolutionary changes of the mammalian skull during the non‐mammaliaform therapsid to mammaliaform transition. First significant changes are found in the main clades of non‐mammaliaform therapsids, which are hypothesized to show a correlated progression of skeletal character evolution leading to higher levels of metabolic activity and homeostatic regulation of the body (Kemp, 2006). The following bony characters with their related soft tissue anatomy (e.g., attaching muscles and/or tendons) are commonly regarded as non‐mammaliaform therapsid synapomorphies, and they are directly and indirectly involved in the evolutionary enhancement of mammalian hearing: the palate, the middle ear, the temporal fenestra (e.g., Maier & Heever, 2002; Kemp, 2005; Romer & Price; Sidor, 2003; Sidor & Hopson, 1998). In general, it is assumed that the evolution and development of the mammalian middle and inner ear are heavily influenced by the expansion of the brain and the formation of the secondary palate in combination with restructuring the lower jaw (Manley, 2010).

During the non‐mammaliaform therapsid to mammaliaform transition, mandibular bones with formally both feeding and hearing functions separated from the lower jaw to become the true mammalian middle ear bones specialized only for hearing. Decoupling of the chewing mechanism and hearing function is a crucial step in mammalian evolution to be able to increase both the chewing efficiency (i.e., food break down in short time) and the hearing ability (i.e., reception of higher frequencies). The incorporation of multiple bony elements into the middle ear increased the hearing sensitivity of mammals, most notably for hearing high‐frequency sounds, a trait that is thought to have benefited the mammalian lineage.

### 3.1 Middle ear perspective

Most significantly, and well documented in the mammalian fossil record, is the separation and size reduction of individual bony elements that later form the mammalian middle ear (Figure 1c). This topic has been the focus of several studies and reviews in the past 15 years (e.g., Anthwal et al., 2013; Ji et al., 2009; Le Maître et al., 2020; Luo, 2011; Luo et al., 2016; Luo & Manley, 2020; Maier & Ruf, 2016; Manley, 2010; Manley, 2017; Norton et al., 2023; Takechi & Kuratani, 2010). In recent years, new fossil findings have brought new perspective on this key evolutionary process (Mao et al., 2020; Mao & Meng, 2020; Schultz et al., 2018; Wang et al., 2019; Wang et al., 2021; Wang & Wang, 2023), which gained significant support from ontogenetic studies that show the recapitulation of crucial steps of the separation of the middle ear elements from the lower jaw. Namely, in developing mammalian embryos, when every future hard tissue structure is still in a cartilaginous state, important stages in development can be observed; yet Ramírez‐Chaves et al., 2016 report a lack of developmental support for the evolutionary mammalian middle ear detachment. For example, the formation of the primary jaw joint between the articular and quadrate bones, with a continuous connection to Meckel's cartilage, which later forms the articulation between the malleus and incus in the middle ear after the breakdown of the Meckel's cartilage connection and functional formation of the secondary jaw joint between the dentary and squamosal (Anthwal et al., 2013). The formation of the primary jaw joint, as well as the breakdown of Meckel's cartilage, significant steps of vertebrate evolution, can also be traced in the chick embryo (Tucker et al., 2004). On a side note, for an excellent overview of the evolution and development of the sauropsid tympanic ear, see Tucker (2017). The homologies of bony elements involved in the evolution of the mammalian middle ear and jaw articulation are well understood and a well‐supported case study in classical embryology (Luo, 2011). According to the “Reichert‐Gaupp theory” (Gaupp, 1911; Reichert, 1837), the cynodont angular, quadrate, and articular bones are homologous to the mammalian ectotympanic ring, malleus, and incus (e.g., Allin, 1975; Allin & Hopson, 1992).

Formation of the mammalian middle ear involves significant changes in the size of the bony elements, starting already in the early diverging non‐mammaliaform therapsids. This mostly involves the stapes and quadrate bones becoming more viable for transmitting vibrations by bone conduction of sound. Interestingly, before size reduction and functional shift to hearing, the stapes had a stabilizing role in the amniote skull during biting (Tucker, 2017). In the early non‐mammaliaform therapsid taxa, the reception of sound occurred most probably via bone conduction along the lower jaw or other bones receiving the sound from the ground, a behavior that is still found in modern squamates, putting the lower jaw to the soil from time to time (Laaß, 2016; Tumarkin, 1968).

The main attachment of the middle ear ossicles in adults of living mammals has shifted from the lower jaw to the basicranium, where they are now sitting in an air‐filled cavity bridging the gap between the external and inner ear by attaching to the tympanic membrane. As this membrane vibrates, the middle ear transmits the vibration to the inner ear, from the malleus, incus to the stapes, the footplate of which is sitting in the oval window of the petrosal bone. The incorporation of the primary jaw joint into the middle ear ossicle chain was only possible due to the evolution of a new way to articulate the upper and lower jaw for feeding, the dentary‐squamosal or secondary jaw joint of mammals (Anthwal & Tucker, 2023). Furthermore, the size reduction of the mammalian middle ear ossicles led to reduced articulation to surrounding structures. A more mobile and flexible attachment facilitates the reception and transmission of high‐frequency sound, a pathway for many modern mammalian taxa to develop ultrasonic hearing. Recent evidence suggests that the evolutionary separation of the middle ear ossicles from the lower jaw probably occurred several times independently in the history of mammals, in monotremes, therians, and allotheria containing multituberculates and euharamiyiids (Luo, 2011; Meng et al., 2018; Urban et al., 2017; Wang et al., 2019). The view of how often this separation occurred depends on the current view of the systematic position of the groups.

The modern mammalian condition, characterized by small middle ear ossicles freely moving inside an air‐filled tympanic cavity in the basicranium is a highly derived evolutionary pattern. How the air‐filled space developed in the different amniote lineages with a tympanic middle ear space is not fully understood, but during the development of the endoderm of the first pharyngeal pouch, it seems to form an extension of the pharynx like a small pocket—the precursor state of the tympanic ear (Buch & Jørgensen, 1964; Schwarzbart, 1958). Supporting this, a study by Thompson and Tucker (2013) shows that the endoderm‐derived epithelium lining the ventral space of the tympanic cavity and the eustachian tube connects the middle ear to the pharynx, while the dorsal space of the tympanic cavity (also known as epitympanic sinus) is lined by the neural crest cells derived epithelium. Thus, the space for the mammalian middle ear cavity has formed from two different developmental derivations. The mammalian middle ear cavity is a unique structure and fundamentally different from the middle ear of non‐mammalian amniotes based on its developmental history and the number of middle ear ossicles and structure of the tympanic membrane (see review by Tucker, 2017). What also remains unsolved is the question of whether the tympanic cavity of monotremes is homologous to that of therian mammals.

In non‐mammalian mammaliaform taxa like the morganucodontan *Morganucodon*, the primary and secondary jaw joints co‐exist in close proximity (Kermack et al., 1981). The appearance of a second jaw articulation is most likely connected to a stabilizing function preventing dislocation during chewing, constraining the movement of the lower jaw and allowing precise occlusion, which is evident from matching wear facets of antagonistic upper and lower teeth (Crompton, 1971; Jäger et al., 2019; Martin et al., 2020). Notably, wear facets indicating precise occlusion appear in concert with the appearance of the double jaw joint in the non‐mammalian mammaliaform fossil record. In the course of mammalian evolution, the primary jaw joint lost its significance during chewing, enabling the functional shift to hearing and constituting the articulation between malleus and incus.

The middle ear homologs of non‐mammalian mammaliaforms are significantly larger than in modern mammals and sit inside the postdentary trough, grooves at the posterior end on the medial side of the dentary, as mentioned above. The ossicles were held inside the grooves of the lower jaw most likely by connective soft tissue with an anterior connection to the thin rod‐like Meckel's cartilage, which is an ossified element and relatively well‐preserved in my specimens of most non‐mammalian mammaliaform taxa (see for example Crompton & Luo, 1993; Meng et al., 2015; Panciroli, Benson, et al. 2019). It is assumed that non‐mammalian mammaliaforms generally were able to receive intermediate frequencies similar to monotremes but lacked the sensitivity to high frequencies known from modern therians (Rosowski, 1992). Different from modern therians, monotremes show not a coiled but a curved cochlea canal with an advanced and organized organ of Corti (similar that of therians). But their upper frequency limit reaches around 15 kHz with best sensitivity at 5 kHz for the platypus and 4–8 kHz in the echidna, according to the obtained threshold curves of audiograms (i.e., for *Ornithorhynchus* see Gates et al., 1974, for *Tachyglossus* see Mills & Shepherd, 2001). The stem mammal groups of the Mesozoic (including multituberculates) with preserved middle and inner ear endocasts probably had an upper frequency limit similar to that of monotremes based on size, mobility of the middle ear ossicles, and cochlea shapes (Manley, 2018; Luo & Manley, 2020: figure 11).

The evolutionary separation of the middle ear elements from the lower jaw starts at the posterior end of the lower jaw by a medial shift of the middle ear ossicles losing the connection to the lower jaw but being suspended by connective tissue still anteriorly connected to Meckel's cartilage. In addition, the middle ear ossicles change their position from being vertical along the dentary to horizontally inclined, nicely preserved in the eutriconodontid *Yanoconodon* (Luo, 2007, Schultz, 2020). Several fossil representatives of stem therians have been discovered in the past 25 years, which show this medial shift and stepwise dislocation of the middle ear ossicles in connection to this progressing size reduction. For example, the zhangeotheriid *Origolestes* even shows first evidence of a separation of the middle ear ossicles from the ossified Meckel's cartilage, but a soft tissue connection probably still existed, and the bony elements start to disconnect (Mao et al., 2020). However, Zhou et al. (2019) posit a connection of the middle ear to Meckel's cartilage for all zhangheotheriids, and an alternative interpretation of the middle ear of *Origolestes* is presented in Luo and Manley (2020).

### 3.2 Inner ear perspective

With the middle ear getting more mobile by size reduction and decoupling from the lower jaw in the lineages leading to modern mammal groups (therians and monotremes), their inner ear adjusted to the finer‐tuned movements in several ways. The stapes sitting inside the oval window of the petrosal bone transmits the vibration of the middle ear to the liquid filling the inner ear. The membranous labyrinth is intricately suspended inside the osseous labyrinth of the petrosal endocast via fibers similar to Sharpey's fibers (Küçük & Abe, 1989), with the two marginal spaces (i.e., scala vestibuli and scala tympani) of the bony cochlear canal filled by perilymph and the membranous space in the middle (i.e., scala media) filled with endolymph. Vibration of the stapedial footplate moves the perilymph fluid, which in turn causes the basilar membrane of the scala media to move. Along the basilar membrane sits the organ of Corti, which functions as a mechanical trigger via bending hair cells. Movements within the fluids initiate the bending of the hair cells, which translate the movement into neural stimuli that are transferred to the brain and are perceived as sound.

In the Mesozoic times, the inner ear of non‐mammalian mammaliaforms did not show the elongated and coiled cochlea like that of therians. The non‐mammalian mammaliaform cochlea is short, straight, or slightly curved but longer than the quite short canals of known non‐mammaliaform therapsid taxa, such as *Pristerodon* or *Cistecephalus* (Benoit et al., 2021; Laaß, 2016). For example, the cochlea canal of the morganucodontan *Morganucodon* shows a straight to slightly curved canal, with a slight extension at the end (recently newly described by Hoffmann et al., 2024 based on CT scans, but was previously described as almost straight, tapering to the tip; Kermack et al., 1981; Graybeal et al., 1989; Luo & Ketten, 1991). Such apical extension at the cochlear end is found in the majority of Mesozoic non‐mammalian mammaliaform taxa (Luo et al., 2016). It seems to disappear from the fossil record with the appearance of therians, coinciding with the innovation of the tribosphenic molar, the full separation of the middle ear ossicles from Meckel's cartilage, and the coiling and first full turn of the cochlear canal.

The apical extension of the cochlea (also termed bulbous ending in other works) is closely linked to the presence of a macula lagena (Figure 1c), which is an additional sensory patch at the end of the cochlear canal already present in amniotic ancestors. The lagena is connected to hearing ability, although it is not really well understood. Two hypotheses exist about the disappearance of the macula lagena in modern mammals (therians and monotremes). The sensory patch was either integrated into the organ of Corti when the evolutionary elongation of the cochlea occurred or simply reduced, because much of the hearing function has been taken over by the organ of Corti (Fritzsch et al., 2013; Schultz et al., 2017). The former hypothesis of integration is complicated as it would involve a spatial reorganization of the macula lagena in relation to the helicotrema, the thin channel connection between the fluid‐filled scala tympani and scala vestibuli (Figure 1d). The therian helicotrema is situated right at the tip of the cochlear canal, while in monotremes and other amniotes, the helicotrema sits in a sub‐apical position (Schultz et al., 2017). While a putative integration of the macula lagena into the organ of Corti would involve substantial structural change, the alternative hypothesis of reduction of this sensory structure would result in the modern mammalian condition in a more straightforward way.

It is not entirely clear when the sensory macula lagena is lost (or integrated into the organ of Corti) in mammalian evolution. The cochlear canal of cladotherians does not show any apical extension or swelling, and it can thus be inferred that in this lineage the lagena is already lost (or integrated). The absence of the apical extension of the bony cochlear canal is therefore a derived condition, like the presence of a primary bony lamina, the presence of a Rosenthal's canal, which are unambiguously recognizable in fossils of Mesozoic cladotherians (Luo et al., 2012, 2016), plus the soft tissue character of the apical position of the helicotrema (Schultz et al., 2017: figure 14).

That the presence of a macula lagena is linked to the presence of an apical extension of the osseous cochlear canal was shown by Schultz et al. (2017), combining histological serial sections and CT scans of the monotreme inner ear to reconstruct the monotreme membranous labyrinth in detail (for both the platypus and echidna). The results show interesting correlations between the osseous cochlear endocast and landmarks of the membranous structure of the cochlea. Although similar in cochlear shape, Schultz et al. (2017) emphasize the important differences in cochlea innervation patterns in monotremes and non‐mammalian mammaliaforms. While monotremes show a tract of small and unordered nerve openings along the dorsal side of the cochlear canal (i.e., tractus foraminosus), most non‐mammalian mammaliaforms described so far have only one large opening on the dorsal side of the cochlear canal for the cochlear nerve to connect to the organ of Corti (see for example Davis et al., 2021; Hoffmann et al., 2024; Panciroli, Schultz, et al., 2019; Schultz et al., 2017; Schultz, Ruf, et al. 2022; Schultz, Schellhorn, et al., 2022).

The ancestral condition of the amniote cochlea includes the presence of the sensory macula lagena, a helicotrema located in a sub‐apical position, a straight to slightly curved cochlear canal with no osseous supporting structures inside (i.e., absence of primary or secondary bony lamina). This plesiomorphic condition is also found in monotremes, although the cochlear canal of monotremes is evidently longer than those of lizards, crocodilians, and turtles (Baird, 1960; Luo & Manley, 2020; Manley, 2018; Schultz et al., 2017). Elongation of the cochlear canal occurred several times and evolved independently in different mammalian lineages: for example, in monotremes (with curved cochlear canals), in therian mammals (with coiled cochlea canals), and in the two known gondwanatherians *Vintana* and *Adalatherium* (which also show elongated, curved and tapering cochlear canals in combination with an advanced innervation pattern including a cribriform plate [Hoffmann et al., 2014; Hoffmann & Kirk, 2020]).

The amniote condition is also inferred for docodontans and morganucodontans, both of which resemble monotremes in characteristics of their osseous cochlear canal except for the different mode of innervation (Hoffmann et al., 2024; Schultz et al., 2017). In amniotes, the basilar papilla (non‐mammalian homolog of the organ of Corti) is innervated via one single opening in the bony cover, similar to what is found in docodontans and morganucodontans and in other Mesozoic taxa like spalacotheroids, multituberculates, and eutriconodontans (Davis et al., 2021; Harper & Rougier, 2019; Hoffmann et al., 2023, 2024; Hurum, 1998; Ladevèze et al., 2010; Luo et al., 2016; Panciroli, Schultz, et al., 2019; Schultz, Ruf, et al., 2022; Schultz, Schellhorn, et al., 2022).

## 4 DISCUSSION

The underlying driving mechanisms for specializations in feeding biomechanics (i.e., mastication, diphyodonty) and adaptation of high‐frequency hearing in the history of mammals are key fields of interest for scientists. Several hypotheses have been discussed in the past, of which the most likely one is linked to insectivory. The ancestors of the non‐mammaliaform therapsid line leading to mammals had predominantly a carnivorous to insectivorous diet often generalized in literature as omnivorous or faunivorous. This conclusion is supported by their dental characteristics, shape and size of the coronoid process, and isotope analysis, as well as by ancestral state reconstruction (Hellert et al., 2023; Hendrickx et al., 2024; Jasinoski et al., 2015; Norton et al., 2020; Rey et al., 2017). For example, the dentition of the epicynodont *Thrinaxodon* suggests that it was a carnivore, focusing its diet mostly on insects, small herbivores, and invertebrates (Brink, 1954; Haughton, 1924). Later in mammalian history, therian mammals most probably developed sensitive hearing for better, targeted detection of insects (Xu et al., 2022) and precise occlusion to break down the chitinous exoskeletons of their prey more effectively (Evans & Sanson, 1998; Spoutil et al., 2010).

Insectivory is also assumed for most non‐mammalian mammaliaform taxa. Many studies show the advantages of the three‐cusped molar pattern for puncturing the exoskeleton of insects (e.g., Evans & Sanson, 1998, 2003; Prinz et al., 2002; Schultz & Martin, 2014). The non‐mammalian mammaliaform three‐cusped pattern is still in function in the tribosphenic molar of therians, with the addition of a distally positioned basin (i.e., talonid) in the lower and a neomorphic protocone in the upper molars. Spoutil et al. (2010) concluded that shearing facets and crests of the tribosphenic molar specifically evolved as an adaptation to insectivory, underlining its universal functionality because the general shape is retained in all extant groups with generalized insectivory (i.e., Ameridelphia, Dasyuromorpha, Afrosoricida, Macroscelidea, Eulipotyphla, Chiroptera, Dermoptera, and Scandentia). It has long been debated whether this tribosphenic mortar‐and‐pistil pattern shows advantages compared to the pre‐tribosphenic three‐cusped embrasure‐shearing pattern. In several studies, the additional breakdown potential of the tribosphenic molar has been revealed (Bhullar et al., 2019; Davis, 2011; Evans & Sanson, 2003; Schultz & Martin, 2014). In addition, the evolutionary plasticity and adaptive potential of the tribosphenic molar possibly have led to the huge diversity of molar shapes in modern mammals, all of which originated from some tribosphenid ancestors.

Bhullar et al. (2019) concluded from their XROMM study on the didelphid *Monodelphis domestica* that an ancestral chewing stroke is conserved in this taxon. The basic pattern they found consists of a simple symmetrical sequence of lower tooth‐row eversion and inversion during jaw opening and closing (by hemimandibular long‐axis rotation) as well as a mortar‐and‐pestle rotational grinding stroke, which the authors hypothesize to be inherited from stem therians along with the tribosphenic molar, and the jaw roll might even be a cladotherian trait. Related to that, Grossnickle (2020b) states that roll‐dominated grinding is not a primitive trait in therians and possibly evolved independently in multiple therian lineages. He further explains that the tribosphenic pattern found in didelphids is a derived character of therians because it is absent in early cladotherians. Grossnickle (2020b) further argues that didelphids possess a broader talonid basin compared to ancestral metatherians and an inflected angular process, which is an apomorphy of Metatheria. Both characters suggest that the roll‐dominated grinding of didelphids rather evolved independently within Metatheria or Marsupialia than being an ancestral pattern. However, as outlined above, the first appearance of several characters near or on the node of Cladotheria likely facilitated the development of precise occlusion and efficient food procession accompanied by the transformation of middle and inner ears that also facilitated enhancement of hearing, including the abilities to hear a broader range of frequencies but also sensitivity to high frequencies.

Beside the evolution of the mammalian chewing function, new fossil findings broadened our view on the ecology of different stem‐line representatives of mammals. As outlined in this review, the dental and skeletomuscular changes in the mammalian masticatory system are likely linked to the evolution of high‐frequency hearing. The disconnection of the postdentary bones from the lower jaw, which later become the mammalian middle ear, facilitated the size reduction of those elements, opening up the possibility to vibrate more sensitively and to transmit a broader spectrum of sounds, received by the elongated, more sensitive cochlea. The lower jaw, in turn—freed from the postdentary elements—can act as a single element, a condition that likely aided the evolution of precise occlusion.

The reconstruction of wing stridulations of Jurassic crickets shows that singing in this group was already a behavior well established by the Middle Jurassic (Bathonian‐Callovian boundary interval, approximately 165 Ma). The study of Gu et al. (2012) reconstructed the paleoacoustics of the low‐frequency musical songs of a katydid cricket, reflecting insect communication already established in the environment of a mid‐Jurassic forest of coniferous trees and giant ferns. The frequency estimated from the number and structure of the wing stridulation of about 6.4 kHz lies well in the frequency range of sound that can be heard by reptilian, amphibian, and early mammalian insectivores. Interestingly, the Gu et al. (2012) study also reveals that modern crickets sing in frequencies of 10–15 kHz, much higher than the songs estimated for the Jurassic katydid. The development of songs of higher frequencies could be hypothesized to be a response to avoid targeted detection by predators (Xu et al., 2022). As discussed above, the best sensitivity recorded for monotremes ranges between 4 and 8 kHz, a hearing spectrum that is also assumed for early mammals based on anatomical similarities (Luo & Manley, 2020: figure 11). As a consequence of the predation pressure on low‐frequency singing insects, it may be that katydid crickets have been under selection pressure to evolve higher pitches of sound. In other words, in order to avoid being detected by predators, crickets (or sound producing insects in general) shifted their singing to higher frequencies, which in turn can be hypothesized to be a possible driver for the evolution of high‐frequency hearing in mammals.

## 5 CONCLUSION

The evolutionary changes of the mammalian skull and lower jaw in the history of mammals involve three major parts that are functionally connected: the lower jaw, middle ear, and inner ear. Structural changes in one part have major impact on the other two. Enhancement of both the feeding and hearing capacity in mammals was likely possible through processes of anatomical disconnection and functional decoupling of the middle ear elements from the lower jaw, a development that is well documented in the Mesozoic part of the mammalian fossil record.

Basal cynodonts like *Thrinaxodon*, evolutionary precursors to mammals, were capable of hearing air‐borne sound directly and via the jaw by bone conduction. Their reception of air‐borne sound was likely adapted to the mid and lower range of frequencies. The same capacity of non‐mammaliaform therapsid ancestors to also hear substrate‐transmitted sounds via the lower jaw was possible by the transmission of ground vibrations along the relatively large angular and surangular bones of the lower jaw, on to the compact articular and quadrate bones, and finally the massive stapes.

The mammalian ability to hear high frequencies of air‐borne sound is a result of the evolutionary process of detaching relatively large, massive middle ear ossicles (as seen in cynodonts) from the feeding system. In addition, mammals reduce the size and mass of the middle ear and elongate the cochlea containing the sensory patches that later become the organized organ of Corti inside the cochlear canal.

## AUTHOR CONTRIBUTIONS

**Julia A. Schultz:** Conceptualization; investigation; writing – original draft; writing – review and editing.
